# Efficient Genetic Transformation and Regeneration of a Farmer-Preferred Cassava Cultivar From Ghana

**DOI:** 10.3389/fpls.2021.668042

**Published:** 2021-05-25

**Authors:** Wilfred Elegba, Emily McCallum, Wilhelm Gruissem, Hervé Vanderschuren

**Affiliations:** ^1^Plant Biotechnology, Institute of Molecular Plant Biology, Department of Biology, ETH Zurich, Zurich, Switzerland; ^2^Biotechnology and Nuclear Agriculture Research Institute, GAEC, Legon, Ghana; ^3^Institute of Biotechnology, National Chung Hsing University, Taichung, Taiwan; ^4^Laboratory of Tropical Crop Improvement, Division of Crop Biotechnics, KU Leuven, Leuven, Belgium; ^5^Plant Genetics, TERRA Research and Teaching Centre, Gembloux Agro BioTech, University of Liège, Gembloux, Belgium

**Keywords:** cassava, organized embryogenic structures, friable embryogenic callus, shoot regeneration, farmer-preferred cultivars, flow cytometry, genetic transformation

## Abstract

Cassava is an important staple crop that provides food and income for about 700 million Africans. Cassava productivity in Africa is limited by viral diseases, mainly cassava mosaic disease (CMD) and cassava brown streak disease (CBSD). Genetic barriers such as high heterozygosity, allopolyploidy, poor seed set, and irregular flowering constrain the development of virus-resistant cassava varieties *via* conventional breeding. Genetic transformation represents a valuable tool to circumvent several challenges associated with the development of virus resistance and other valuable agronomic traits in cassava. The implementation of genetic transformation in many local African cultivars is limited either by the difficulty to produce friable embryogenic callus (FEC), low transformation, and/or regeneration efficiencies. Here, we report the successful induction of organized embryogenic structures (OES) in 11 farmer-preferred cultivars locally grown in Ghana. The production of high quality FEC from one local cultivar, ADI 001, facilitated its genetic transformation with high shoot regeneration and selection efficiency, comparable to the model cassava cultivar 60444. We show that using flow cytometry for analysis of nuclear ploidy in FEC tissues prior to genetic transformation ensures the selection of genetically uniform FEC tissue for transformation. The high percentage of single insertion events in transgenic lines indicates the suitability of the ADI 001 cultivar for the introduction of virus resistance and other useful agronomic traits into the farmer-preferred cassava germplasm in Ghana and Africa.

## Introduction

Nearly 850 million people living in the tropics depend on the starchy storage roots of cassava (*Manihot esulenta* Crantz) as an important source of carbohydrates in their diet or as raw material in several industries (FAO, 2013). In Africa, cassava is cultivated mainly by smallholder farmers because the crop produces appreciable yields under a wide range of environmental conditions (Ceballos et al., 2004; El-Sharkawy, 2006; Burns et al., 2010). Cassava production in Africa is constrained by weeds, drought, pests, and most importantly, viral diseases (Patil and Fauquet, 2009; Vandegeer et al., 2013; Legg et al., 2015; Ekeleme et al., 2019; Orek et al., 2020). Cassava mosaic disease (CMD) and cassava brown streak disease (CBSD) are the two most prevalent viral diseases limiting cassava production in Africa (Legg et al., 2011; Patil et al., 2015; Rey and Vanderschuren, 2017). Conventional breeding approaches to develop virus resistant cassava varieties are lengthy and constrained by high genome heterozygosity, irregular flowering, and low seed set (Jennings and Iglesias, 2002; Kawano, 2003; Ceballos et al., 2004; Nassar, 2010; Bull et al., 2017). Despite these limitations, molecular breeding of cassava has led to the identification of different sources of resistance such as *CMD1*, which is conferred by polygenic recessive genes (Fregene et al., 1997), the single dominant monogenic gene, *CMD2* (Akano et al., 2002; Fregene et al., 2006; Rabbi et al., 2014), and *CMD3* (Okogbenin et al., 2012).

Genetic transformation can complement traditional or molecular breeding approaches in developing cassava varieties with engineered improved traits such as virus resistance, amylose-free starch, increased micronutrient content, reduced cyanogen content, and micronutrient composition (Siritunga and Sayre, 2003; Jorgensen et al., 2005; Zhang et al., 2005; Vanderschuren et al., 2009, 2012, 2014; Liu et al., 2011; Li et al., 2015; Bull et al., 2018; Narayanan et al., 2019, 2020). Genetic transformation is also a key step in the development of transgene-free genome edited cassava as recently demonstrated by the transformation of cassava with an expression cassette containing genes for Cas9, guide-RNA, and *Arabidopsis* FLOWERING LOCUS T for early flowering, which was subsequently segregated out by a genetic cross under greenhouse conditions (Bull et al., 2018; Zaidi et al., 2019).

Genetic transformation of cassava requires suitable target tissues for successful recovery of transformed plants. To date, somatic embryo cotyledons (Luong et al., 1995; Li et al., 1996; Zhang et al., 2000; Ihemere et al., 2006), embryogenic suspension cultures (Schöpke et al., 1993, 1996; González et al., 1998; Schreuder et al., 2001), and friable embryogenic callus (Taylor et al., 1996; Bull et al., 2009; Zainuddin et al., 2012; Chetty et al., 2013; Nyaboga et al., 2013) have been transformed successfully. However, challenges such as genotype-dependent production of friable embryogenic callus (FEC), low regeneration rates from transformed calli or low conversion of somatic embryos into plants have also been reported (Schöpke et al., 1996; Baba et al., 2008; Liu et al., 2011; Zainuddin et al., 2012; Nyaboga et al., 2013; Lentz et al., 2018).

Friable embryogenic calli are now mostly used for cassava transformation because the tissue is suitable for large production of independent transgenic events with various cultivars, and particularly the model cultivar 60444 (Schreuder et al., 2001; Bull et al., 2009; Taylor et al., 2012; Chetty et al., 2013). FEC tissue reduces the risk of generating chimeric plants because regenerating plantlets originate from individual transformed cells (Taylor et al., 1996, 2012; González et al., 1998; Bull et al., 2009; Zainuddin et al., 2012; Nyaboga et al., 2013, 2015). A major challenge of FEC-based cassava transformation is the generation of FEC suitable for transformation of local cultivars or landraces. Induction of FEC tissue is genotype-dependent and requires optimization for each cultivar (Raemakers et al., 2001; Rossin and Rey, 2011; Zainuddin et al., 2012; Chetty et al., 2013; Nyaboga et al., 2013, 2015; Lentz et al., 2018). Moreover, potential genome instability and changes in gene expression following embryogenesis (Ma et al., 2015), or the loss of resistance to CMD during tissue culture (Beyene et al., 2016a; Chauhan et al., 2018), necessitate protocol modifications for different cultivars.

Induction of high quality FECs using the protocol established by Bull et al. (2009) has been successful for the African farmer- or industry-preferred cassava landraces TME3, TME7, TME204, TME14, T200, Ebwanatereka, Kibandameno, and Serere (Bull et al., 2009; Zainuddin et al., 2012; Chetty et al., 2013; Nyaboga et al., 2013, 2015). Following initial efforts to adapt the protocol for the transformation of farmer-preferred genotypes mainly from Africa (Taylor et al., 2012; Nyaboga et al., 2013; Chauhan et al., 2015; Beyene et al., 2016b), expanding transformation to other farmer-preferred landraces across Africa is needed for adoption of transgenic technologies. It is important to enable transgenic technologies for each region of Africa by establishing protocols for cultivars that have been selected by farmers for improved traits and adaptation to local environments (Fregene et al., 2000; Vanderschuren, 2012). The induction of high quality FEC tissues suitable for transformation from a broad range of African cassava genotypes is critical for the development and deployment of cultivars with resistance to cassava mosaic geminiviruses (CMGs) and cassava brown streak viruses (CBSVs) across Africa. More importantly, it is key to establish robust genetic transformation protocols that can be implemented in African laboratories (Vanderschuren, 2012; Chetty et al., 2013; Nyaboga et al., 2013; Ojola et al., 2018; Walsh et al., 2019).

Here, we report the successful induction of organized embryogenic structures (OES) in 11 farmer-preferred cultivars locally grown in Ghana. The production of high quality FEC suitable for genetic transformation was achieved in one local cultivar, ADI 001. The efficiencies of FEC induction, genetic transformation and regeneration in transformed tissues of ADI 001 were comparable to the model cultivar, 60444. Our protocol for genetic transformation of a local cassava cultivar can help to expand transformation capacity to other locally adapted cassava cultivars in Africa and to facilitate crop biotechnology capacities in African laboratories.

## Materials and Methods

### Plant Material

Eleven cassava genotypes representative of the varieties cultivated by farmers in Ghana (Afisiafi, Ankra, ADI 001, Bosomnsia, Dagarti, IFAD, Megyewontem, Nkabom, Santum, Tomfa, and Tuaka) were obtained from the *in vitro* cassava library of the Biotechnology and Nuclear Agriculture Research Institute (BNARI), Ghana. The model cultivar, 60444 was obtained from the *in vitro* germplasm collection of the Plant Biotechnology Laboratory at ETH Zurich. All plant material was maintained *in vitro* on cassava basic medium (CBM) media containing Murashige and Skoog (MS) basal salts with vitamins (Duchefa Biochemie, RV Haarlem, Netherlands), 20 g L^−1^ sucrose (Roth, Switzerland), 2 mM CuSO_4_ (Sigma-Aldrich, Munich, Germany), 3 g L^−1^ Gelrite (Duchefa Biochemie, RV Haarlem, Netherlands), and 50 μg ml^−1^ carbenicillin (Duchefa Biochemie, RV Haarlem, Netherlands) at 28°C, 16/8 h light/dark regime in growth cabinets.

### Induction and Proliferation of Organized Embryogenic Structures and Friable Embryogenic Callus

Organized embryogenic structures and FEC induction was initiated in Ghanaian cassava cultivars according to the protocol established by Bull et al. (2009) as briefly summarized here. For OES induction, stem cuttings (ca. 5 mm long) from 4 weeks old *in vitro* plants were placed horizontally on axillary bud enlargement medium (CAM) for 2–4 days (Zhang and Gruissem, 2004; Bull et al., 2009; Zainuddin et al., 2012). Enlarged axillary buds were isolated with a sterile syringe needle and transferred to OES induction medium (CIM). Plates were kept in the dark for 14 days and explants transferred to fresh CIM plates. OES induction and multiplication was carried out on CAM containing MS basal medium containing 6-benzylaminopurne (BAP; 10 mg L^−1^). For multiplication, OES were transferred to MS basal medium containing 12 mg L^−1^ 4-amino-3, 5, 6-trichloro-2-pyridinecarboxylic acid (picloram). Thirty-six explants per cultivar were assessed on picloram-containing medium and experiments were repeated four times.

For induction of FECs, OES generated from the selected cultivars on CIM media were transferred to Gresshoff and Doy (GD) medium (Duchefa Biochemie, RV Haarlem, Netherlands; Gresshoff and Doy, 1974) containing 12 mg L^−1^ picloram. OES were transferred to fresh GD media every 21 days (up to a maximum of six transfer stages; GD1–GD6). Similarly, any FEC material formed from OES was transferred every 21 days to fresh GD media for further multiplication and selection of quality friable embryogenic tissue.

To improve FEC induction in farmer-preferred cassava cultivars, we replaced the auxin picloram in the CIM media with another synthetic auxin, 2,4-dichlorophenoxyacetic acid (2, 4-D; Duchefa Biochemie, RV Haarlem, Netherlands) at a concentration of 8 mg L^−1^. After 4 days of incubation on CAM medium, swollen axillary buds were excised and transferred to CIM media supplemented with 8 mg L^−1^ 2,4-D. Any OES tissues formed were transferred to GD medium supplemented with 8 mg L^−1^ 2,4-D. OES were transferred to fresh GD media up to a maximum of six transfer stages; GD1–GD6. This experiment was repeated twice with a minimum of 48 explants per cultivar (six plates with eight explants per plate) used for each experiment. In both experiments, OES tissues from picloram and 2,4-D were kept separate at the different stages (CAM, CIM, and GD stages).

### Ploidy Analysis of FECs Induced From Ghanaian Cassava Cultivar, ADI 001

An Otto buffer protocol with 4',6-diamidino-2-phenylindole (DAPI) staining adapted for cassava FEC tissues was used to measure ploidy levels. Specifically, a small sample of FECs was collected from at least three clusters per plate and three plates for each cultivar. FECs were transferred into wells of a 96-well Micronic plate (Lelystad, Netherlands). Two TOMY SUB-30 beads (Adolf Kuhner, Switzerland) and 300 μl of Otto I buffer (0.1 M citric acid, 0.5% Tween-20) was added to each well. Tissues were ground using a Retsch mill MM301 (Retsch GmbH, Switzerland) for 15 s at 12.5 revolutions per minute (rpm). As much supernatant as possible was transferred to a 25–30 μM UNIFILTER® plate (Whatman®) and centrifuged immediately for 10 s at 1000 rpm. Around 100 μl of filtrate was transferred to a 96-well plate and 100 μl Otto II buffer (0.6 M hydrogen phosphate, with 12 μg ml^−1^ DAPI added immediately before use) was added to each well. Samples were pipetted gently up and down to mix thoroughly and then analyzed with a CytoFLEXS flow cytometer (Beckman Coulter).

### Regeneration of Wild-Type ADI 001 FECs

The regeneration potential of wild-type FECs induced from ADI 001 and 60444 was measured using five plates per cultivar consisting of six FEC clusters. FEC clusters were transferred to embryo maturation and regeneration medium (MSN) containing 1 mg L^−1^ naphthalene acetic acid (NAA) and 250 mg L^−1^ carbenicillin (MSN + C250) as previously described by Bull et al. (2009). FEC clusters were transferred to fresh MSN media every 10–14 days for four successive cycles (MSN1–MSN4). Maturing cotyledon embryos at MSN 4 stage were counted and transferred to cassava shoot elongation medium (CEM) containing 100 mg L^−1^ carbenicillin (CEM + C100). After 3 weeks on CEM, well-developed shoots were counted and transferred to CBM containing 50 mg L^−1^ carbenicillin for shoot establishment. The regeneration potential was measured as the total number of plants regenerated over 80 days.

### Binary Vectors and *Agrobacterium* Inoculum

A binary vector, pRNAi-dsAC1 described by Vanderschuren et al. (2009), hereafter referred to as “pCAMBIA-based vector,” was transformed into *Agrobacterium tumefaciens* strain LBA4404 *via* electroporation. Agrobacterium suspension cultures were prepared according to Bull et al. (2009). Briefly, single Agrobacterium colonies carrying the aforementioned plasmid was transferred from Luria broth (LB) plates containing kanamycin (50 mg L^−1^), rifampicin (50 mg L^−1^), and streptomycin (100 mg L^−1^) and used to inoculate 5 ml liquid LB cultures containing the same antibiotics. Cultures were grown overnight at 28°C on a shaker at 200 rpm until an optical density (OD)_600_ between 0.7 and 1 was reached. Around 5 ml of the starter culture was used to inoculate 25 ml of LB culture containing antibiotics and grown overnight at 28°C and 200 rpm until OD_600_ = 0.7–1. Suspensions were centrifuged at 4,000 *g* for 10 min at room temperature, the supernatant discarded and pellet washed with GD liquid medium. This process was repeated and the bacterial pellet re-suspended in GD medium and diluted to an OD_600_ = 0.5. Acetosyringone was added to the suspension to a final concentration of 200 μM.

### Genetic Transformation, Regeneration, and Screening of Transgenic Plants

For each cultivar, five FEC plates (each plate contains six FEC clusters) were inoculated with *Agrobacterium* carrying the pCAMBIA-based binary vector. FECs were co-cultivated with *Agrobacterium* suspension for 3 days at 24°C with 16/8 h light/dark photoperiod. Co-cultivated FECs were gently scraped from plates into a 50 ml sterile Falcon tube (Sarstedt, Germany) and washed several times with GD solution containing 500 mg L^−1^ carbenicillin until the supernatant was clear. FECs were transferred to mesh and briefly dried on filter paper before transfer to solid GD media containing 250 mg L^−1^ carbenicillin (C250) for 4 days. Thereafter, FECs were transferred with the mesh to fresh GD medium containing C250 and 5 mg L^−1^ hygromycin. After 7 days, FECs were transferred to GD media containing C250 and increasing concentrations of hygromycin (8 and 15 mg L^−1^) at weekly intervals.

For regeneration, matured FEC tissues were transferred to regeneration medium (MSN + C250) containing 15 mg L^−1^ hygromycin. Weekly transfer of FEC tissues to freshly prepared regeneration medium was carried out for six successive cycles (MSN1–MSN6). Developing green cotyledons at each MSN stage were counted and transferred to CEM+C100 media. After 3 weeks, well-developed shoots were excised from cotyledons and transferred to CBM + C50 for shoot establishment.

Screening of fully developed plantlets was performed by transferring stem cuttings to CBM media containing 50 mg L^−1^ carbenicillin and 10 mg L^−1^ hygromycin (rooting test). The total number of plants positive for the rooting test was counted. Regeneration in transformed tissues was measured as the total number of plants regenerated over 100 days.

### Molecular Characterization of Transgenic Lines

Cassava genomic DNA was extracted from flash frozen leaves using the modified cetyltrimethyl ammonium bromide (CTAB) method (Doyle, 1990). PCR analysis was carried out to confirm integration of a 152 bp region of the AC1 gene in regenerated plants of ADI 001 and 60444. PCR thermal cycle conditions used were as follows: Initial denaturation for 20 s at 95°C, 35 cycles of denaturation for 3 s at 95°C, annealing for 15 s at 60°C, extension for 30 s at 72°C and final extension for 5 min at 72°C. PCR products were resolved on 1% w/v agarose gel and images recorded using a Gel iX20 imager gel documentation system (INTAS science imaging system, Goettingen, Germany).

For Southern blot analysis, aliquots of 15 μg DNA were digested with the restriction enzyme, HindIII (Thermo Scientific, Switzerland) and products separated on a 1% w/v agarose gel. Transfer of digested products to nylon membrane (Hybond-N+; Amersham Pharmacia Biotech) was performed overnight and hybridization with probes specific for the hygromycin phosphotransferase II (hptII) gene was carried out (Vanderschuren et al., 2009; Zainuddin et al., 2012). Probes were prepared by PCR amplification of a 978 base pair (bp) amplicon of the hygromycin gene and DIG-dUTP labeled using a PCR DIG probe synthesis kit (Roche Diagnostics GmbH, Germany) according to the manufacturer’s instructions. Primers used for PCR screening and amplification of the probe for Southern blot are listed in Supplementary Table 1.

### Data Analysis

Data for all parameters except plant regeneration efficiencies in wild-type and transgenic 60444 and ADI 001 FEC tissues was analyzed using GraphPad Prism version 7 (San Diego, United States). Shoot regeneration efficiencies were analyzed using Minitab version 18 (Pennsylvania, United States). All data was subjected to ANOVA and statistically significant results at the 5% level were eith2er compared with Tukey’s or Šidák’s multiple comparisons tests.

## Results and Discussion

### Selection of Farmer-Preferred Cultivars and Induction of Organized Embryogenic Structures

**Figure 1:**
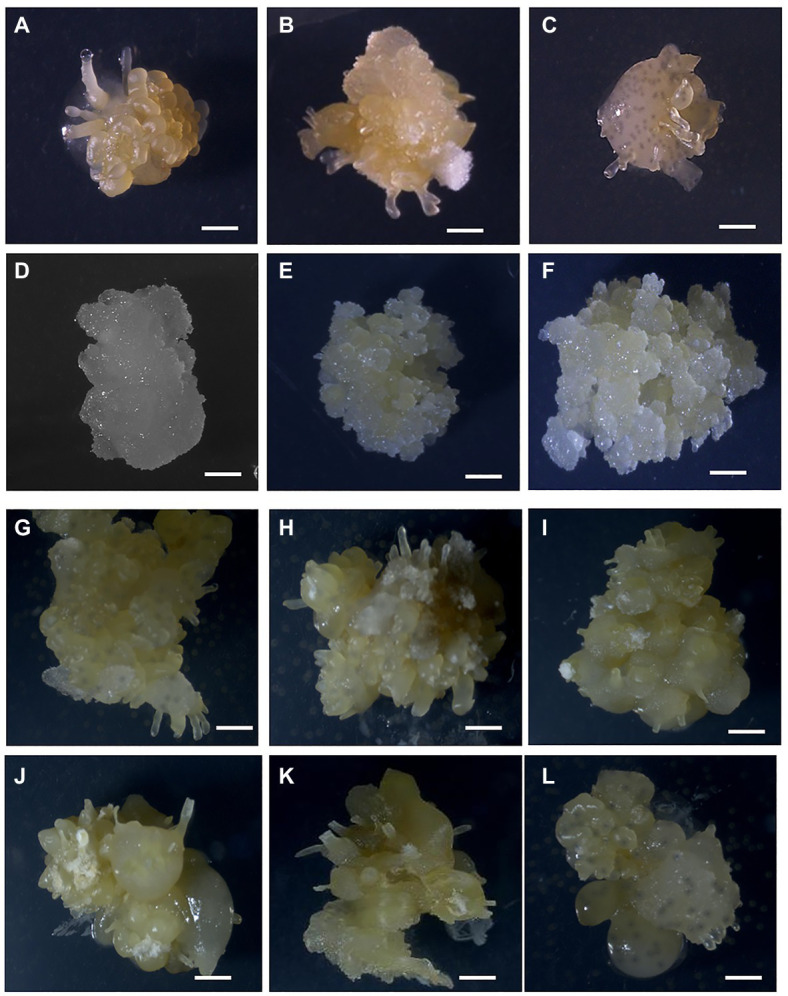
Formation of OES and FEC in Ghanaian cassava cultivars. OES production on CIM media containing picloram (12 mg L^−1^; **A**) 60444, **(B)** Santum, **(C)** ADI 001, **(D)** IFAD. Proliferating FEC tissues in cultivars **(E)** 60444 and **(F)** ADI 001. OES production on CIM media containing 2,4-D (8 mg L^−1^; **G**), 60444 **(H)** ADI 001, **(I)** Ankra, **(J)** IFAD, **(K)** Megyewontem, and **(L)** Nkabom. Bar represents 2 mm.

In Africa, preference for traits such as high yield, good cooking quality, early maturity, weed suppression, and resistance to pest and disease influence cultivar adoption by farmers (Hahn et al., 1989; Manu-Aduening et al., 2006). We selected 11 cassava cultivars or landraces popular with farmers in Ghana (Table 1). These cultivars have varying degrees of susceptibility to CMD and CBSD (Elegba et al., 2020). We screened the selected cultivars for production of OES, a critical initial step required for the induction of FEC and subsequent genetic transformation. In cassava, induction of somatic embryogenesis (SE) is genotype-dependent and some cultivars, and in particular African cultivars, lose embryogenic competence prior to formation of somatic embryos (Konan et al., 1994; Atehnkeng et al., 2006). Variations in cultivar response to SE have been reported for South American cultivars (Ihemere, 2003). SE has been previously established for two Ghanaian cultivars, ADI 001 and Nkabom, using young leaf lobe explants (Danso et al., 2010). We used axillary meristems as starting material because of the higher efficiency of OES production and reduced production of non-embryogenic callus compared to leaf lobe explants (Taylor et al., 2012; Nyaboga et al., 2015). After 14 days on CIM media, OES were induced from axillary buds in all 11 Ghanaian cassava cultivars on picloram-containing media (Figures 1A–D). OES production in the model cultivar 60444 was significantly higher (*p* ≤ 0.05) compared to Ghanaian cassava cultivars except for Santum (*p* = 0.06; Table 1; Supplementary Table 2). In contrast, there was no significant difference in OES production frequency between 60444 and Ghanaian cassava cultivars on 2,4-D-containing media (Table 1; Supplementary Table 3). With the exception of Tuaka and Bosomnsia that failed to form OES on 2,4-D-containing media in two independent experiments, we noticed that OES production was significantly higher (*p* ≤ 0.05) on 2,4-D-containing medium compared to picloram-containing medium in 60444 and all Ghanaian cassava cultivars tested except Santum (Table 1; Supplementary Table 4). However, on 2,4-D containing media, OES tissues produced in Ghanaian cassava cultivars and 60444 looked darker in color compared with picloram-derived tissues by the third cycle on CIM (Figures 1G–L). The highest OES production frequency on picloram and 2,4-D-containing media was recorded in Santum (55%) and Megyewontem (92%), respectively (Table 1). This successful production of OES from axillary bud explants in locally adapted cassava cultivars preferred by farmers in Ghana is important for induction of FECs and subsequent genetic transformation.

**Table 1:** Agronomic traits, qualities, and average frequency of organized embryogenic structure (OES) production in selected Ghanaian cassava cultivars.

Cassava cultivar	Agronomic traits	Status	OES frequency (%) ± SE^a^	
			Picloram^b^	2,4-D^c^
60444	Model cultivar	Released variety	74 ± 3.42^a^	86 ± 3.54^a^
ADI 001	Early maturing (6–8 months), mealiness	Landrace	52 ± 4.99^bc^	90 ± 0.00^ac^
Santum	High starch content, mealiness	Released variety	55 ± 5.32^a^	56 ± 3.54^ad^
IFAD	High dry matter (30%), high yield (30–35 t/ha), CMD tolerant	Released variety	35 ± 3.30^b^	81 ± 0.00^a^
Megyewontem	Early maturing (6–8 months)	Landrace	37 ± 2.99^bc^	92 ± 4.95^ae^
Nkabom	High dry matter (32%), high yield (28–32 t/ha), CMD tolerant	Released variety	17 ± 2.16^bd^	78 ± 7.78^a^
Tomfa	Dry matter (29%), starch content (58%), mealiness	Released variety	42 ± 5.29^be^	84 ± 4.95^a^
Ankra	High dry matter (34%), high starch content (68%), mealiness	Released variety	31 ± 1.63^bd^	84 ± 7.78^a^
Dagarti	High dry matter (37%), high starch content (65%), CMD tolerant	Released variety	36 ± 2.94^b^	75 ± 14.14^a^
Afisiafi	High dry matter (32%), high yield (28–35 t/ha), CMD tolerant	Released variety	37 ± 5.56^be^	79 ± 2.83^a^
Tuaka	Early maturing (6–8 months), dry matter (23%)	Landrace	12 ± 2.22^bce^	0^b^
Bosomnsia	Early maturing (6–8 months), mealiness	Landrace	14 ± 4.35^b^	0^b^

a OES production frequency calculated as ratio of OES clusters/total number of explants expressed as a percentage.

b OES frequency on picloram shows means of four independent experiments ± SE.

c OES frequency on 2,4-D shows means of two independent experiments ± SE. (Tukey’s multiple comparison test, *p* ≤ 0.05).

d,e represent significant differences in OES frequencies on picloram and 2,4-D between cultivars (at 95 or 99% confidence interval difference).

### FEC Induction in Ghanaian Cassava Cultivar ADI 001

Using the FEC induction protocol optimized for the model cultivar, 60444, we first tested the 11 Ghanaian farmer-preferred cassava cultivars for FEC induction and multiplication from OES obtained on picloram-containing medium. After 6 weeks on GD medium containing picloram at 12 mg L^−1^, OES tissues of 60444 and the Ghanaian cassava cultivar ADI 001 produced FECs (Figures 1E,F). However, OES tissues in the remaining 10 Ghanaian cultivars failed to form FECs on GD media. After 8 weeks on GD medium, OES became covered by growth of whitish-colored non-embryogenic callus tissue (Figures 1C,D). This observation was confirmed in four independent experiments in which only OES from ADI 001 and 60444 produced FECs.

Friable embryogenic callus induced from OES in ADI 001 were pale yellow in color and showed a good rate of proliferation similar to FEC from 60444 (Figures 1E,F). A major bottleneck for genetic transformation of farmer-preferred cassava cultivars or landraces is the failure to generate FECs from OES as we also found for the selected Ghanaian cultivars and similar to what has been reported for other cassava landraces cultivated in West Africa (Raemakers et al., 2001; Hankoua et al., 2006). Therefore, the protocol reported by Bull et al. (2009) that has been successfully used for FEC production of several cassava cultivars requires further optimization to increase the success of FEC induction in a broad range of cassava cultivars and landraces.

We next tested if OES obtained on 2,4-D-containing medium could improve FEC induction in Ghanaian cassava cultivars (Table 1). We used 8 mg L^−1^ 2,4-D as a previously reported optimal concentration for induction of high frequency somatic embryos (Danso and Ford-Lloyd, 2002; Danso et al., 2010). However, we observed a decrease in growth and number of OES by the second cycle on GD medium (GD2) for FEC induction in 60444 and the Ghanaian cultivars we tested (Supplementary Table 5). Only 60444 OES tissues converted to FECs, although at low frequency (Supplementary Table 5). After 8 weeks on GD medium, OES tissues of Ghanaian cultivars turned dark yellow or brown in color and converted to non-embryogenic structures (Figures 1G–L). Therefore, addition of 2,4-D in the FEC induction medium (CIM) seems to favor continuous non-OES cell proliferation instead of conversion to FEC (Taylor et al., 1996). In contrast, as shown above and reported earlier, picloram in combination with GD medium is efficient in the conversion of embryogenic tissues to FECs in cassava (Taylor et al., 1996; Bull et al., 2009; Zainuddin et al., 2012; Chetty et al., 2013; Nyaboga et al., 2013). The exact mechanism of picloram on FEC induction in cassava is not clearly understood. However, initiation of somatic embryogenesis involves a weakening of the cell to cell interaction gradient that coordinates bipolar embryo development (Pretova and Williams, 1986) and it appears picloram is more effective compared to 2,4-D at stimulating the weakening process. The upregulation of cell cycle related genes and several genes involved in brassinosteriod signaling and jasmonate (JA) metabolism, such as polysaccharide hydrolase, are likely to influence cell wall modifications (Nowack et al., 2006; Elhiti et al., 2013; Ma et al., 2015).

### Nuclear Ploidy of 7-Month-Old FECs in ADI 001 and 60444 Remains Stable

Continuous passage of tissues through *in vitro* culture over time can cause changes such as chromosomal rearrangements or increase in chromosome number and ploidy level, which can result in somaclonal variation and potential phenotype changes in regenerated plants (Kaeppler et al., 2000; Thorpe, 2006; Miguel and Marum, 2011; Stroud et al., 2013; Ma et al., 2015). Morphological changes and decreased regeneration potential have been associated with FECs that have been maintained for long periods of time (i.e., over 6 months) in liquid suspension culture (Raemakers et al., 2001; Ma et al., 2015). Therefore, it is important to include quality control steps during the production and regeneration of embryogenic cassava cells. We assessed the stability of nuclear ploidy of FECs induced from ADI 001 and 60044 using flow cytometry of FEC tissue after 10 successive cycles of subculture (approximately 7-months) on GD medium containing picloram (12 mg L^−1^). Nuclei from a tomato leaf sample were used as a DNA reference standard. The tomato nuclear genome is 960 Mb and therefore similar in size to the cassava nuclear genome (750 Mb), which minimizes errors resulting from sampling or instrument variation (Vindeløv et al., 1983; Bagwell et al., 1989). Nuclei from a cassava leaf sample of 60444 were calibrated against the reference tomato standard to confirm diploid genome status.

**Figure 2:**
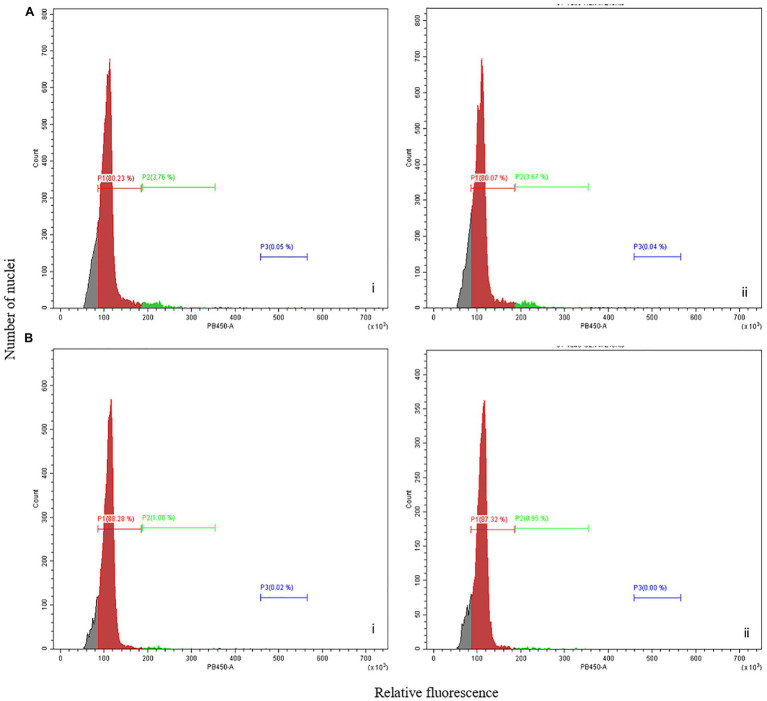
Flow cytometry analysis of picloram-induced FECs. Flow cytometry analysis showing relative fluorescence intensity of FECs induced in cassava cultivar **(A)** ADI 001 and **(B)** 60444. Biological replicates, *n* = 2 (indicated by i and ii) are shown for panels (A,B). Each histogram shows the nuclear DNA level for each clump of FEC sampled, *n* = 3.

Analysis of the flow cytometry profiles of nuclei isolated from several independent FEC clumps of ADI 001 showed one dominant peak equivalent to the diploid 60444 leaf nuclei reference (Figure 2A). Similarly, the ploidy profile of FECs induced from 60444 exhibited a single diploid peak (Figure 2B). These results indicate that FECs induced from ADI 001 and 60444 have mainly nuclei with diploid genomes even after 10 cycles of subculture on picloram-containing medium.

Building robust platforms for genetic transformation of a broad range of cassava cultivars requires routine induction of OES and FEC, and regeneration of transformed plants through somatic embryogenesis. We suggest that integrating flow cytometry analysis of nuclear genome ploidy into the FEC induction and genetic transformation pipeline ensures reliable FEC ploidy stability at the early stages of FEC multiplication for transformation. This avoids the regeneration of plants from polyploid somatic embryos that produce not true-to-type plants with phenotypic changes such as thicker stems, variation in leaf anatomy, longer petiole, and internodal distances (Awoleye et al., 1994; Taylor et al., 2004; Nassar et al., 2008), which is often manifested only during later stages of plant regeneration and growth.

### Regeneration of Plants From Picloram-Induced Wild-Type FECs

Genotypes also determine the regeneration efficiency of cassava cultivars (Raemakers et al., 2001; Ihemere, 2003; Hankoua et al., 2005; Atehnkeng et al., 2006; Zainuddin et al., 2012; Chetty et al., 2013; Nyaboga et al., 2013). Therefore, testing the shoot regeneration efficiency of FECs produced from local landraces or farmer-preferred cassava cultivars is important to ensure the production of whole plants from explants is feasible.

**Figure 3:**
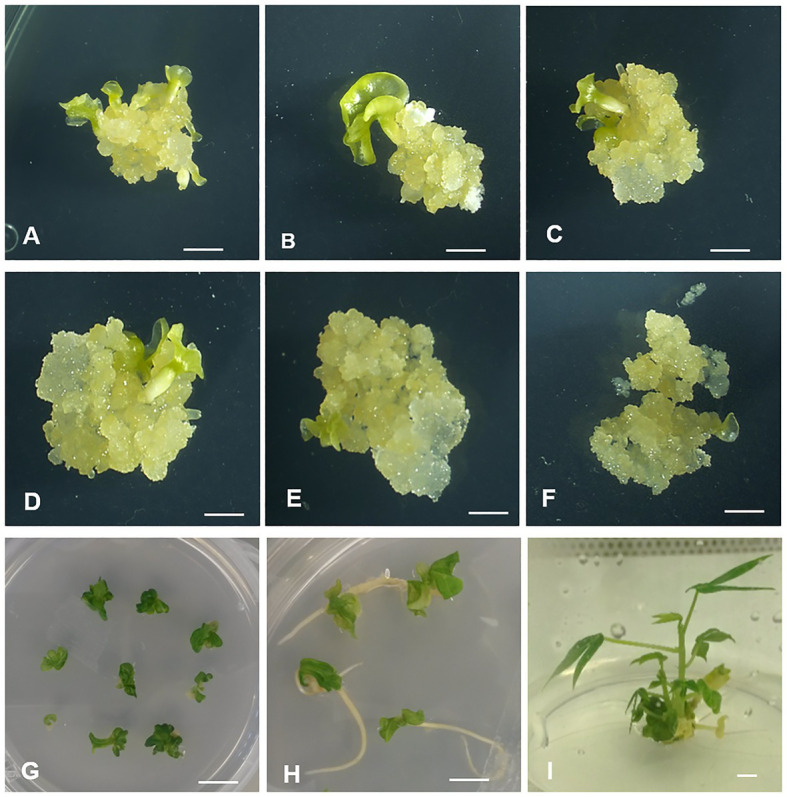
Whole plant regeneration from picloram-induced wild-type FECs of ADI 001 and 60444. Embryo emergence in ADI **(A–C)** and 60444 **(D–F)**. Cotyledon development **(G,H)** and whole plant regeneration **(I)** in wild-type ADI 001 FECs. Bars represents 2 mm **(A–H)** and 1 mm **(I)**.

We evaluated the regeneration potential of wild-type ADI 001 FECs in comparison to 60444 (Figure 3). Cotyledon regeneration in ADI 001 (Figures 3A–C) was observed after 10 days on MSN1 media compared to 12 days for 60444 (Figures 3D–F). The number of embryos progressing into cotyledon stages in ADI 001 and 60444 increased significantly (*p* ≤ 0.05) after 20 days on embryo maturation and regeneration medium (Supplementary Figure 1; Supplementary Table 6). At 40 days after transfer of embryos to maturation medium, there was no significant difference (*p* = 0.824) in the total number of regenerated cotyledons from wild-type FECs of ADI 001 (Figures 3G,H) and 60444 (Table 2; Supplementary Table 7). We recovered from 30 FEC clumps 160 cotyledon-stage embryos from ADI 001 and 150 cotyledon-stage embryos from 60444. The number of whole plants that regenerated from wild-type cotyledon-stage embryos was similar (*p* = 0.729) between 60444 and ADI 001 (Supplementary Table 7). Plants regenerated from ADI 001 FECs looked normal (Figure 3I). Together, our results indicate that the Ghanaian cassava cultivar ADI 001 performs equally well as 60444 with respect to FEC induction and regeneration. The high plant regeneration efficiency that we achieved in ADI 001 (above 60%) indicates that this cultivar may also allow for high transformation capacity.

**Table 2:** Plant regeneration from wild-type ADI 001 and 60444 friable embryogenic callus (FEC) tissues.

Cultivar	No. of regenerated cotyledons^a^	No. of regenerated plants	Plant regeneration efficiency^b^ (%)
60444	150^a^	107^a^	71
ADI 001	160^a^	97^a^	61

a Total number of cotyledons regenerating on embryo maturation media [MSN+C250 + 1 mg L^−1^ naphthalene acetic acid (NAA)] from 30 FEC clusters (five plates; six clusters per plate).

b Total number of plants regenerated on cassava basic medium (CBM) media/total number of regenerated cotyledons. Values in a column followed by the same letters are not significantly different from each other at *p* ≤ 0.05 (Tukey’s pairwise comparison test).

### Efficient Genetic Transformation of Ghanaian Farmer-Preferred Cassava Cultivar ADI 001

Following the evaluation of the ADI 001 regeneration capacity, we tested the transformability of this Ghanaian cultivar. To determine the transformation efficiency of ADI 001, we transformed 60444 and ADI 001 FEC tissues using a pCAMBIA-based vector carrying a hairpin construct against the GMS AC1 gene that had been previously transformed successfully (Vanderschuren et al., 2009). After five weekly transfers of FEC tissues on embryo maturation and regeneration media containing 250 mg L^−1^ carbenicillin and 15 mg L^−1^ hygromycin, the total number of regenerated cotyledons and plants from transgenic FECs of ADI 001 was not significantly different compared to 60444 (Table 3; Supplementary Table 8). Plant regeneration efficiency in the local cultivar, ADI 001 (58%) was comparable to 60444 (65%; Table 3).

**Table 3:** Plant regeneration efficiency in transgenic ADI 001 and 60444 FEC tissues.

Cultivar	No. of regenerated cotyledons^a^	No. of regenerated plants	Plant regeneration efficiency^b^ (%)
60444	88^a^	57^a^	65
ADI 001	134^a^	78^a^	58

a Total number of cotyledons regenerating on embryo maturation media (MSN +C250 + 15 mg L^−1^ hygromycin) from 30 FEC clusters (five plates; six clusters per plate).

b Total number of plants regenerated on CBM media (prior to rooting test)/total number of regenerated cotyledons. Values in a column followed by the same letters are not significantly different from each other at *p* ≤ 0.05 (Tukey’s pairwise comparison test).

Rapid and efficient screening of regenerating potential in transgenic plantlets is an important step to identify plants with a T-DNA insertion in the genome, thereby reducing the number of transgenic plants for subsequent molecular characterization. To assess the efficiency of Agrobacterium-mediated transformation of the ADI 001 genotype and to identify transformed plantlets, we used a rooting test, which is efficient for identification of transgenic cassava plants (Zhang and Puonti-Kaerlas, 2000; Bull et al., 2009; Zainuddin et al., 2012). In the rooting test, hygromycin-resistant transgenic cassava shoots produce roots and leaves on hygromycin-containing media (CBM + C50 + H10), indicating successful integration of the T-DNA carrying the *hptII* gene into the genome. Almost 90 % of the regenerated ADI 001 plantlets were positive for the rooting test compared to 56 % of the 60444 regenerated plantlets (Supplementary Table 9). Although, we only performed and characterized the transformation with a single batch of ADI 001 FEC, the good selection efficiency of transgenic ADI 001 plantlets suggests that this cultivar is amenable to genetic transformation. In order to confirm integration of the T-DNA cassette into the cassava genome, we screened by PCR a selected number of ADI 001 plants that were positive in the rooting test (Supplementary Figure 2).

We subsequently determined the number of independent transgenic events using Southern blot analysis on rooting test positive transgenic ADI 001 and 60444 plantlets. Transgene integration was detected in all transgenic lines screened for ADI 001 and 60444 (Supplementary Figure 3). Based on the hybridization pattern, all of the 17 transgenic lines screened for ADI 001 and 60444 were independent transgenic events. Because plants with low transgene copy numbers (ideally a single insert) are preferred for use in further characterization and subsequent confined field trials, we calculated the percentage of single insertion events as the number of lines containing a single insert of the transgene. In 60444, 47 % of the lines screened by Southern blot had a single transgene insert compared to 65% of lines screened for ADI 001 (Supplementary Table 10).

## Conclusion

Genetic improvement of cassava requires the establishment of robust protocols for routine transformation, especially for local cultivars preferred by African farmers. In the present study, we report successful implementation of the protocol reported by Bull et al. (2009) for induction of high quality FECs in an elite local cassava cultivar from Ghana. Efficient genetic transformation and high plant regeneration from transformed FECs of ADI 001 was comparable to the model cultivar, 60444. However, as observed in the present study, the transfer of transformation capacity to local cultivars with farmer- and industry-preferred traits is limited due to high genotypic influence on the embryogenic process. Thus, to unlock the potential of important African cassava cultivars, it is critical to further work on optimization of the protocol for routine production of FEC tissues amenable to genetic transformation, on a cultivar-by-cultivar basis. Optimization of culture conditions or media composition has led to an improvement in FEC induction and subsequent transformation of several African and Brazilian cultivars (Zainuddin et al., 2012; Nyaboga et al., 2013, 2015; Lentz et al., 2018).

More importantly, transfer of transgenic technologies to laboratories in Africa, such as the Biotechnology and Nuclear Agriculture Research Institute (BNARI) in Ghana, through capacity building as has been accomplished through this work is critical for encouraging and sustaining adoption of transgenic technologies in Africa (Ezezika et al., 2012; Vanderschuren, 2012). The presence of appropriately trained personnel is critical to the establishment and maintenance of transformation platforms for crop improvement in Africa. The establishment of cassava transformation platforms in sub-Saharan Africa opens up opportunities for improvement of other important traits such as extension of shelf life, nutrient content, pest resistance, climate-change resilience as well as serve as a basis for the rapid exploitation of CRISPR-based technologies to enhance food security.

## Data Availability Statement

The original contributions presented in the study are included in the article/Supplementary Material, further inquiries can be directed to the corresponding authors.

## Author Contributions

HV, WE, EM, and WG conceived and designed the experiments and reviewed and edited the manuscript. WE and EM performed the experiments. WE, HV, and WG analyzed the data. All authors contributed to the article and approved the submitted version.

### Conflict of Interest

The authors declare that the research was conducted in the absence of any commercial or financial relationships that could be construed as a potential conflict of interest.
