# Supplementary material for: Efficient Genetic Transformation and Regeneration of a Farmer-Preferred Cassava Cultivar From Ghana
Source: Front Plant Sci. 2021 May 25;12:668042. doi: 10.3389/fpls.2021.668042 (PMC8204248; doi:10.3389/fpls.2021.668042)
Supplement: Supplementary file 1 [file Data_Sheet_1.docx]

Supplementary Material

**Supplementary Table 1**. Primer sequences used for PCR and Southern blot screening of ADI 001 and 60444 transgenic plants

| **Primer name** | **Primer sequence** | **Target sequence** | **Amplicon size (bp)** |
| --- | --- | --- | --- |
| dsAC1:FP | FP-5’ -CCACTATCGGCGAGTACTTCTACACAG -3’ | ACMV AC1 sequence |  |
| dsAC1:RP | RP- 5’- GCCTGAACTCACCGCGACGTCTGTC -3’ |  | 152 |
| Probe 1 fwd | FP-5’ -CCACTATCGGCGAGTACTTCTACACAG -3’ | hygromycin resistance gene (hptII) | 978 |
| Probe 1 rev | RP- 5’- GCCTGAACTCACCGCGACGTCTGTC -3’ |  |  |

**Supplementary Table 2.** Two-way ANOVA comparing OES production on picloram-containing medium in eleven farmer-preferred cassava cultivars from Ghana

| **Source of Variation** | **% of total variation** | **P value** | **P value summary** | **Significance** |  |
| --- | --- | --- | --- | --- | --- |
| Interaction | 5.904 | 0.0102 | * | Yes |  |
| Cultivars | 26.74 | <0.0001 | **** | Yes |  |
| picloram | 51.88 | <0.0001 | **** | Yes |  |
|  |  |  |  |  |  |
| **ANOVA table** | **SS** | **DF** | **MS** | **F (DFn, DFd)** | **P value** |
| Interaction | 1826 | 11 | 166.0 | F (11, 72) = 2.497 | P=0.0102 |
| Cultivars | 8267 | 11 | 751.6 | F (11, 72) = 11.31 | P<0.0001 |
| picloram | 16043 | 1 | 16043 | F (1, 72) = 241.4 | P<0.0001 |
| Residual | 4785 | 72 | 66.45 |  |  |

Interaction = interaction between picloram and cultivars; cultivars = eleven Ghanaian cassava cultivars tested; residual = variation among replicates not related to systematic differences between picloram and cultivars; SS = sum of squares; DF= degrees of freedom; MS = mean squares; F (DFn, DFd) = F ratio (MS value divided by residual)

**Supplementary Table 3.** Two-way ANOVA comparing OES production on 2,4-D-containing medium in eleven farmer-preferred cassava cultivars from Ghana

| **Source of Variation** | **% of total variation** | **P value** | **P value summary** | **Significance** |  |
| --- | --- | --- | --- | --- | --- |
| Interaction | 5.050 | 0.0231 | * | Yes |  |
| Cultivars | 71.30 | <0.0001 | **** | Yes |  |
| 2,4-D | 19.46 | <0.0001 | **** | Yes |  |
|  |  |  |  |  |  |
| **ANOVA table** | **SS** | **DF** | **MS** | **F (DFn, DFd)** | **P value** |
| Interaction | 2333 | 11 | 212.2 | F (11, 24) = 2.630 | P=0.0231 |
| Cultivars | 32939 | 11 | 2994 | F (11, 24) = 37.13 | P<0.0001 |
| 2,4-D | 8993 | 1 | 8993 | F (1, 24) = 111.5 | P<0.0001 |
| Residual | 1936 | 24 | 80.65 |  |  |

Interaction = interaction between 2,4-D and cultivars; cultivars = eleven Ghanaian cassava cultivars tested; residual = variation among replicates not related to systematic differences between 2,4-D and cultivars; SS = sum of squares; DF= degrees of freedom; MS = mean squares; F (DFn, DFd) = F ratio (MS value divided by residual)

**Supplementary Table 4.** Two-way ANOVA comparing OES production frequencies in eleven farmer-preferred cassava cultivars on picloram and 2,4-D-containing media

| **Source of Variation** | **% of total variation** | **P value** | **P value summary** | **Significance** |  |
| --- | --- | --- | --- | --- | --- |
| Interaction | 33.74 | <0.0001 | **** | Yes |  |
| Cultivar | 41.51 | <0.0001 | **** | Yes |  |
| Auxins | 23.12 | <0.0001 | **** | Yes |  |
|  |  |  |  |  |  |
| **ANOVA table** | **SS** | **DF** | **MS** | **F (DFn, DFd)** | **P value** |
| Interaction | 16703 | 11 | 1518 | F (11, 48) = 12.89 | P<0.0001 |
| Cultivars | 20552 | 11 | 1868 | F (11, 48) = 15.86 | P<0.0001 |
| Auxins | 11449 | 1 | 11449 | F (1, 48) = 97.19 | P<0.0001 |
| Residual | 5655 | 48 | 117.8 |  |  |

Interaction = interaction between picloram and cultivars; cultivars = eleven Ghanaian cassava cultivars tested; Auxins = picloram and 2,4-D; residual = variation among replicates not related to systematic differences between picloram and 2,4-D; SS = sum of squares; DF= degrees of freedom; MS = mean squares; F (DFn, DFd) = F ratio (MS value divided by residual)

**Supplementary Figure 1.** Regeneration of cotyledon-stage embryos from wild-type FECs in ADI 001 and 60444 at different stages on embryo maturation and regeneration medium (MSN +C250). FEC clusters were transferred to fresh MSN media every 10 -14 days.

**Supplementary Table 5.** Average OES and FEC production frequencies in eleven farmer-preferred cassava cultivars from Ghana on 2,4-D- supplemented medium

| Cultivar | OES frequency^a^ (%) | | OES frequency (%) on GD ^b^ | | FEC production frequency^c^ (%) ± SE |
| --- | --- | --- | --- | --- | --- |
|  | **1** | **2** | **1** | **2** |  |
| 60444 | 54 | 49 | 47 | 10 | 23 ± 5.66 |
| ADI 001 | 65 | 65 | 63 | 30 | 0 |
| Santum | 16 | 11 | 11 | 4 | 0 |
| IFAD | 58 | 58 | 57 | 36 | 0 |
| Megyewontem | 70 | 63 | 63 | 54 | 0 |
| Nkabom | 52 | 41 | 40 | 24 | 0 |
| Tomfa | 54 | 47 | 47 | 36 | 0 |
| Ankra | 46 | 35 | 35 | 33 | 0 |
| Dagarti | 60 | 35 | 35 | 9 | 0 |
| Afisiafi | 40 | 36 | 36 | 18 | 0 |
| Tuaka | 0 | 0 | 0 | 0 | 0 |
| Bosomnsia | 0 | 0 | 0 | 0 | 0 |

^a^OES frequency from a minimum of 48 axillary bud explants.

^b^OES frequency at third cycle on GD medium

^c^FEC production frequency calculated as total number of FEC clusters/total number of OES explants expressed as a percentage.

FEC frequency shows mean ± SE of 2 independent experiments.

**Supplementary Table 6.** Two-way ANOVA comparing regeneration of wild-type cotyledon-stage embryos in ADI 001 and 60444 FECs at different stages on MSN media

| **Source of Variation** | **% of total variation** | **P value** | **P value summary** | **Significance** |  |
| --- | --- | --- | --- | --- | --- |
| Interaction | 0.4898 | 0.1426 | ns | No |  |
| media | 96.82 | <0.0001 | **** | Yes |  |
| cultivar | 0.003038 | 0.8504 | ns | No |  |
|  |  |  |  |  |  |
| **ANOVA table** | **SS** | **DF** | **MS** | **F (DFn, DFd)** | **P value** |
| Interaction | 36.28 | 3 | 12.09 | F (3, 32) = 1.942 | P=0.1426 |
| media | 7170 | 3 | 2390 | F (3, 32) = 383.9 | P<0.0001 |
| cultivar | 0.2250 | 1 | 0.2250 | F (1, 32) = 0.03614 | P=0.8504 |
| Residual | 199.2 | 32 | 6.225 |  |  |

Interaction = interaction between media and cultivars; media = different stages of MSN media ( MSN 1 – 4); cultivar = ADI 001 and 60444; residual = variation among replicates not related to systematic differences between media and cultivars; SS = sum of squares; DF= degrees of freedom; MS = mean squares; F (DFn, DFd) = F ratio (MS value divided by residual)

**Supplementary Table 7.** One-way ANOVA table comparing regeneration of cotyledons and plants in wild-type ADI 001 and 60444 FEC tissues

|  | **Source** | **DF** | **Adj SS** | **Adj MS** | **F-Value** | **P-Value** |
| --- | --- | --- | --- | --- | --- | --- |
| Cotyledons | cultivar | 1 | 12.50 | 12.50 | 0.05 | 0.824 |
|  | Error | 6 | 1393.00 | 232.17 |  |  |
|  | Total | 7 | 1405.50 |  |  |  |
| Plants | cultivar | 1 | 12.50 | 12.50 | 0.13 | 0.729 |
|  | Error | 6 | 569.50 | 94.92 |  |  |
|  | Total | 7 | 582.00 |  |  |  |

DF= degrees of freedom; Adj SS = adjusted sum of squares; Adj MS = adjusted mean squares; F-value = ratio of 2 MS square values

**Supplementary Table 8.** One-way ANOVA table comparing cotyledon and plant regeneration in transgenic ADI 001 and 60444 FEC tissues

|  | **Source** | **DF** | **Adj SS** | **Adj MS** | **F-Value** | **P-Value** |
| --- | --- | --- | --- | --- | --- | --- |
| Cotyledons | cultivar | 1 | 264.5 | 264.50 | 4.74 | 0.072 |
|  | Error | 6 | 335.0 | 55.83 |  |  |
|  | Total | 7 | 599.5 |  |  |  |
| Plants | cultivar | 1 | 55.13 | 55.13 | 2.07 | 0.200 |
|  | Error | 6 | 159.75 | 26.62 |  |  |
|  | Total | 7 | 214.88 |  |  |  |

DF= degrees of freedom; Adj SS = adjusted sum of squares; Adj MS = adjusted mean squares; F-value = ratio of 2 MS square values

**Supplementary Table 9.** Selection efficiency of transgenic plants regenerated in ADI 001 and 60444

| **Cultivar** | **Plants regenerated^a^** | **Rooting test positive^b^** | **Selection efficiency^c^ (%)** |
| --- | --- | --- | --- |
| 60444 | 95 | 53 | 56 |
| ADI 001 | 123 | 109 | 89 |

^a^Total number of plants regenerated from 30 FEC clumps (5 plates) on used for rooting test

^b^Total number of plants that formed roots on selection medium (CBM + C50 +H10)

^c^Total number of plants positive for rooting test/ total number of plants used for rooting test

Only plants displaying normal phenotypes were selected for rooting tests.


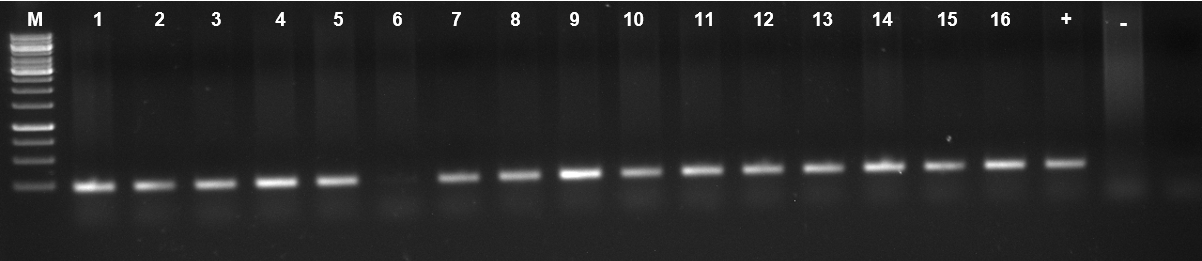


**Supplementary Figure 2.** PCR screening of selected transgenic ADI 001 plants using AC1-specific primers

Lane M: Gene ruler 100bp DNA ladder, lanes 1-16: selected transgenic plants of ADI 001 positive for rooting test, lane **+**: transgenic 60444 line (positive control), lane **-** : wild-type 60444 (negative control). Primer used for detection of 152 bp amplicon (see supplementary table S1).


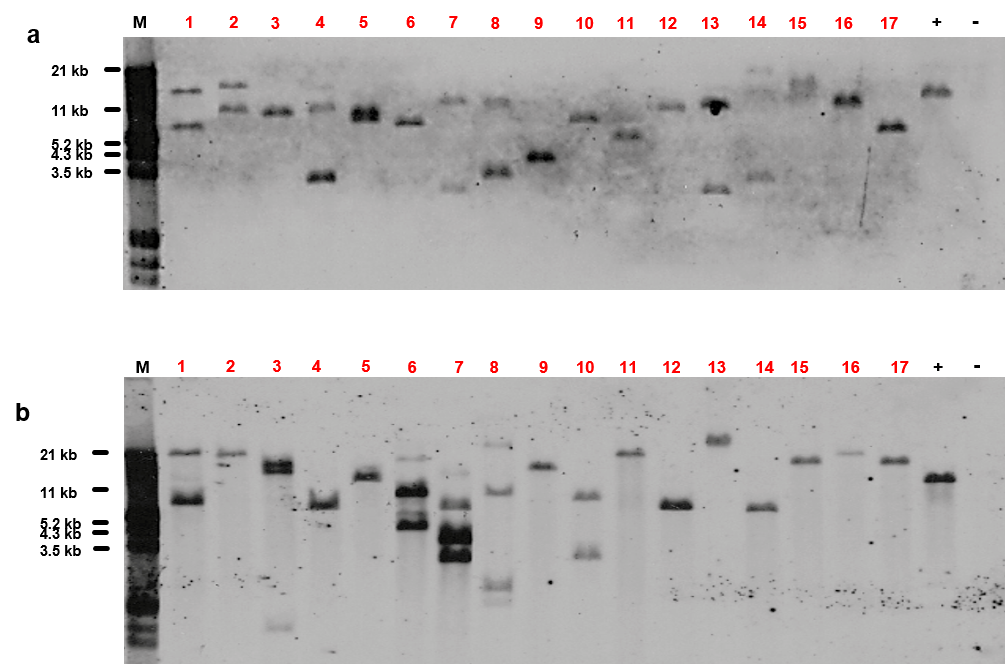


**Supplementary Figure 3.** Southern blot analysis of transgenic ADI 001 and 60444 plants.

DNA isolated from (**a**) ADI 001 and (**b**) 60444 plants, which were positive in the rooting test. Genomic DNA was digested with *Hind*III and hybridized with DIG-labelled probe for the *hptII* gene. Independent ADI 001 and 60444 transgenic lines are indicated in red font.

**Supplementary Table 10.** Transformation efficiency (single copy lines only) of transgenic plants regenerated in ADI 001 and 60444

| **Cultivar** | **No. of lines screened** | **No. of single insertion lines^a^** | **Single insertion events ^b^ (%)** |
| --- | --- | --- | --- |
| 60444 | 17 | 8 | 47 |
| ADI 001 | 17 | 11 | 65 |

^a^Total number of lines with single insertion of the transgene; ^b^Total number of lines with single insertion of transgene / total number of lines screened
